# Likely causal effects of insulin resistance and IGF-1 bioaction on childhood and adult adiposity: a Mendelian randomization study

**DOI:** 10.1038/s41366-024-01605-4

**Published:** 2024-08-22

**Authors:** Duaa I. Olwi, Lena R. Kaisinger, Katherine A. Kentistou, Marc Vaudel, Stasa Stankovic, Pål R. Njølstad, Stefan Johansson, John R. B. Perry, Felix R. Day, Ken K. Ong

**Affiliations:** 1 MRC Epidemiology Unit, Institute of Metabolic Science, University of Cambridge, Cambridge, CB2 0QQ UK; 2 King Abdullah International Medical Research Center, Jeddah, Saudi Arabia; 3 King Saud bin Abdulaziz University for Health Sciences, Jeddah, Saudi Arabia; 4 Mohn Center for Diabetes Precision Medicine, Department of Clinical Science, University of Bergen, NO-5020, Bergen, Norway; 5 Department of Genetics and Bioinformatics, Health Data and Digitalization, Norwegian Institute of Public Health, NO-0213, Oslo, Norway; 6 Department of Pediatrics and Adolescents, Haukeland University Hospital, NO-5021, Bergen, Norway; 7 Department of Medical Genetics, Haukeland University Hospital, NO-5021, Bergen, Norway; 8 Metabolic Research Laboratory, Institute of Metabolic Science, University of Cambridge, Cambridge, CB2 0QQ UK; 9 Department of Paediatrics, University of Cambridge, Cambridge, UK

**Keywords:** Risk factors, Epidemiology

## Abstract

**Background:**

Circulating insulin and insulin-like growth factor-1 (IGF-1) concentrations are positively correlated with adiposity. However, the causal effects of insulin and IGF-1 on adiposity are unclear.

**Methods:**

We performed two-sample Mendelian randomization analyses to estimate the likely causal effects of fasting insulin and IGF-1 on relative childhood adiposity and adult body mass index (BMI). To improve accuracy and biological interpretation, we applied Steiger filtering (to avoid reverse causality) and ‘biological effect’ filtering of fasting insulin and IGF-1 associated variants.

**Results:**

Fasting insulin-increasing alleles (35 variants also associated with *higher* fasting glucose, indicative of insulin resistance) were associated with lower relative childhood adiposity (*P* = 3.8 × 10^−3^) and lower adult BMI (*P* = 1.4 × 10^−5^). IGF-1-increasing alleles also associated with *taller* childhood height (351 variants indicative of greater IGF-1 bioaction) showed no association with relative childhood adiposity (*P* = 0.077) or adult BMI (*P* = 0.562). Conversely, IGF-1-increasing alleles also associated with *shorter* childhood height (306 variants indicative of IGF-1 resistance) were associated with lower relative childhood adiposity (*P* = 6.7 × 10^−3^), but effects on adult BMI were inconclusive.

**Conclusions:**

Genetic causal modelling indicates negative effects of insulin resistance on childhood and adult adiposity, and negative effects of IGF-1 resistance on childhood adiposity. Our findings demonstrate the need to distinguish between bioaction and resistance when modelling variants associated with biomarker concentrations.

## Introduction

Obesity presents a major health challenge throughout the life-course, with childhood obesity persisting into adulthood and extending its implications beyond youth [1]. Both childhood and adult obesity are associated with changes in the levels of circulating insulin and insulin-like growth factor-1 (IGF-1) [2]. Investigating the potential causal effects of these biomarkers on obesity may help in designing effective strategies for preventing and managing obesity.

It is known that exogenous insulin therapy and medications such as sulfonylureas or glinides, which are used to enhance the body’s natural insulin production in patients with type 2 diabetes, confer increased weight gain [3]. An observational analysis of data from longitudinal studies and clinical trials found that increases in fasting insulin lead to subsequent increases in BMI [4]. However, it is unclear if this relationship reflects a positive effect of insulin on BMI as, conversely, hyperinsulinaemia is typically interpreted as a marker of insulin resistance [5].

The observed phenotypic association between IGF-1 and obesity is inconsistent. Studies have reported that the relationship between IGF-1 and BMI is negative [6, 7], positive [8] or non-linear [9, 10]. Additionally, the relationship between IGF-1 and adiposity may be influenced by its role as a positive marker of growth hormone axis activity, which potentially affects statural growth more than adipose tissue accumulation [11].

Due to the heterogeneous observations surrounding fasting insulin and IGF-1, the causal nature of these biomarkers on adiposity remains unclear. The identification of genetic variants by genome-wide association studies (GWAS) that are robustly associated with these hormones provides an opportunity to test for causal associations using Mendelian randomization (MR) [12]. Within the MR framework, the random allocation of alleles at conception helps eliminate possible confounding due to environmental factors. Additionally, methods such as Steiger filtering make this more robust by reducing the likelihood of reverse causality. In this study, we used ‘biological effect’ filtering to distinguish whether variants associated with higher biomarker concentrations are indicative of greater biomarker action or greater biomarker resistance. Using a two-sample MR approach, we aimed to examine the causal associations of fasting insulin and IGF-1 on relative childhood adiposity and adult body mass index (BMI).

## Methods

Two-sample univariate MR analyses were performed to assess the effect between genetically predicted fasting insulin and IGF-1 on childhood and adult obesity. We conducted the analyses on 444,345 European individuals from the UK Biobank participants with self-reported relative childhood adiposity [13]. We verified any findings in a smaller sample of 39,725 participants with objectively measured childhood BMI from the Early Growth Genetics (EGG) consortium [14]. Additionally, we tested the associations with adult BMI using data from 456,426 European individuals from the UK Biobank [15].

MR utilizes GWAS summary statistics data to test the likely causal association between an exposure and outcome of interest. It relies on three key assumptions: (a) the instrumental variants are associated with the exposure; (b) the instrumental variants are not associated with confounders; and (c) the instrumental variants only influence the outcome via the exposure of interest. We selected as instrumental variants those single nucleotide polymorphisms (SNPs) that reached genome-wide significant threshold (*P* < 5.0 × 10^−8^) for circulating fasting insulin or IGF-1 concentrations. In all analyses, the variants were aligned to designate the exposure-increasing allele as the effect allele. For variants that were not present in the outcome data, a highly correlated proxy was selected (within 1 Mb and *r*^*2*^ > 0.7; Supplementary Table 1) using a linkage-disequilibrium panel from a random subsample of individuals in the UK Biobank study with self-identified and genetically confirmed white European ancestry.

MR assumes a consistent and linear relationship, but a higher biomarker level may indicate enhanced biomarker action or increased biomarker resistance. To distinguish between these contrasting effects, we applied ‘biological effect’ filtering in our MR models. This method involved filtering genetic variants based on their individual effect on a third phenotype, which serves as an established indicator of that biomarker’s action.

### Instrumental variables

#### Fasting insulin

**Fig. 1:**
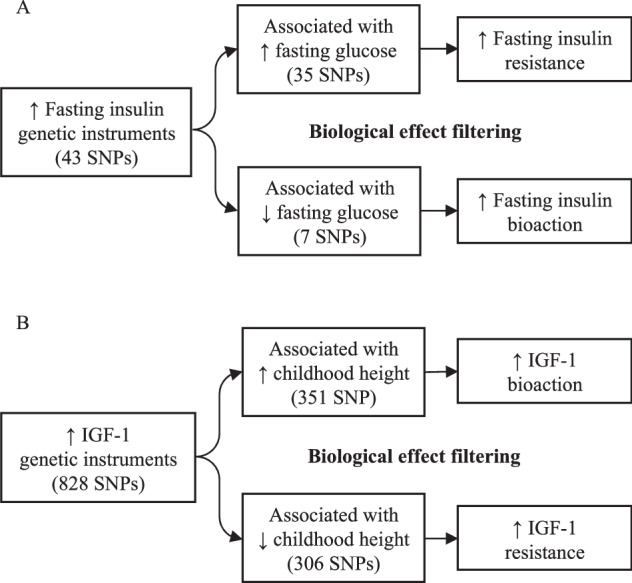
Stratification of biomarker-associated genetic instruments by their biological effects on established downstream traits. **A** Fasting insulin associated genetic instruments were filtered by their direction of effect on fasting glucose. **B** IGF-1 associated genetic instruments were filtered by their direction of effect on childhood height. IGF-1 insulin-like growth factor-1, SNPs single nucleotide polymorphisms.

Genetic instruments for fasting insulin concentration (log_n_ pmol/L, in models adjusted for BMI) were based on a European GWAS meta-analysis of approximately 150,000 individuals without diabetes, which reported 43 independent signals [16]. As higher insulin concentrations typically reflect greater insulin resistance [17], they can also reflect greater insulin secretion and bioaction [18]. Therefore, we stratified the insulin genetic instruments based on their effect on fasting glucose concentration, as the established role of insulin is to lower circulating glucose [16] (Fig. 1). Genetic data for fasting glucose (BMI adjusted) was based on a European GWAS meta-analysis of 200,621 individuals [16].

Of the 43 insulin-increasing alleles, 35 were also directionally associated with higher fasting glucose (indicating insulin resistance), while 7 were directionally related to lower fasting glucose (indicating greater insulin secretion and bioaction). For the remaining one fasting insulin associated variants, there was no available proxy.

#### IGF-1

Genetic instruments for circulating IGF-1 concentration (nmol/L) were based on a European GWAS of 428,525 European participants from the UK Biobank, which we resolved to 828 independent signals [19]. Higher IGF-1 concentrations typically reflect higher IGF-1 secretion and bioaction; but can also reflect IGF-1 resistance (for example, due to damaging mutations in the IGF-1 receptor gene, *IGF1R* [20]). Therefore, we stratified the IGF-1 genetic instruments by their effect on childhood height, as the established role of IGF-1 is to increase height [11] (Fig. 1). GWAS data for childhood height at age 7 was based on 36,102 children from the Norwegian Mother, Father and Child Cohort Study (MoBa) [21].

Of the 828 IGF-1-increasing alleles, 351 were also directionally related to taller childhood height (indicating greater IGF-1 secretion and bioaction), while 306 were directionally related to shorter childhood height (indicating IGF-1 resistance). For the remaining 171 IGF-1 associated variants, there were no suitable proxies (*r*^*2*^ > 0.7) for childhood height in the MoBa data.

### Outcome variables

#### Relative childhood adiposity

GWAS data for relative childhood adiposity in the UK Biobank study were modelled as a primary outcome. This was assessed in 444,345 European participants in response to the question “*When you were 10 years old, compared to average would you describe yourself as thinner, plumper, or about average?*” (UK Biobank field 1687). The GWAS model treated responses as a linear variable (thinner/average/plumper) [13].

To provide confirmation of the results obtained using relative childhood adiposity, we also used data on measured childhood BMI (in standardized units) from a much smaller GWAS meta-analysis. These data from the EGG consortium were based on a trans-ancestral meta-analysis GWAS of BMI in 39,620 children aged between 2 and 18 years [14].

#### Adult BMI

GWAS data for adult BMI was based on 456,426 European-ancestry participants from the UK Biobank using data from the first assessment centre visit (UK Biobank field 21001) [15].

### Statistical analysis

#### MR models

Our primary analysis was the inverse-variance weighted (IVW) MR model with biological effect and Steiger filtering. Steiger filtering reduces reverse causality by removing instrumental variable SNPs if they have a stronger effect on the outcome than on the exposure (Supplementary Table 2). We performed sensitivity analyses that control for genetic pleiotropy, including MR-Egger, Weighted Median (WM), and Penalized Weighted Median (PWM) MR models. Additionally, we used the MR-Egger intercept *P* < 0.05, *I*^2^ statistic, Cochran’s *Q*-derived *P* value, funnel and dosage plots to assess evidence of balanced and unbalanced pleiotropy. When MR-Egger intercept is significant (*P* < 0.05), we considered the IVW model to be invalid. All analyses were performed using R (version 3.5.1) and a *P* < 0.0125 was considered statistically significant (calculated as 0.05 / (2 exposures * 2 outcomes)).

## Results

### Estimated effect of fasting insulin on adiposity

#### Fasting insulin (non-stratified SNPs)

**Fig. 2:**
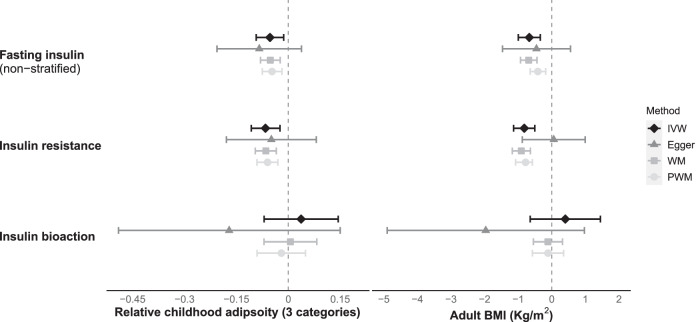
Estimated effects of fasting insulin (log_n_ pmol/L) on relative childhood adiposity and adult BMI. Insulin resistance was represented by fasting insulin-increasing alleles also associated with higher fasting glucose. Insulin bioaction was represented by fasting insulin-increasing alleles also associated with lower fasting glucose. Estimates are from random-effects inverse-variance weighted analyses with Steiger filtering. BMI body mass index, IVW inverse-variance weighted, WM Weighted Median, PWM Penalized Weighted Median.

Overall, genetically-predicted higher fasting insulin concentrations were associated with lower relative childhood adiposity (β = −0.053 categories per log_n_ pmol/L; *P* = 1.2 × 10^−2^) and lower adult BMI (β = −0.669 Kg/m^2^ per log_n_ pmol/L; *P* = 2.7 × 10^−4^) (Fig. 2 and Supplementary Table 3). Additional MR models using measured childhood BMI confirmed the relationship between higher fasting insulin and lower childhood BMI (β = −0.11 SD per log_n_ pmol/L; *P* = 4.4 × 10^−2^) (Supplementary Fig. 1 and Supplementary Table 3).

#### Insulin resistance

Fasting insulin-increasing alleles that were also associated with higher fasting glucose (indicative of insulin resistance) were associated with lower relative childhood adiposity (β = −0.066 category per log_n_ pmol/L; *P* = 3.8 × 10^−3^) and lower adult BMI (β = −0.819 Kg/m^2^ per log_n_ pmol/L; *P* = 1.4 × 10^−5^) (Fig. 2 and Supplementary Table 4). Additional MR models using measured childhood BMI confirmed the relationship between insulin resistance and lower childhood BMI (β = −0.126 SD per log_n_ pmol/L; *P* = 3.2 × 10^−2^) (Supplementary Fig. 1 and Supplementary Table 4).

#### Insulin bioaction

Conversely, insulin-increasing alleles that were also associated with lower fasting glucose (indicators of insulin secretion and bioaction) showed no association with relative childhood adiposity nor with adult BMI (*P* = 0.521 and *P* = 0.478, respectively) (Fig. 2 and Supplementary Table 4). Additional MR models using measured childhood BMI showed no association between insulin bioaction and childhood BMI (*P* = 0.985) (Supplementary Fig. 1 and Supplementary Table 4).

### Estimated effect of IGF-1 on adiposity

#### IGF-1 (non-stratified SNPs)

**Fig. 3:**
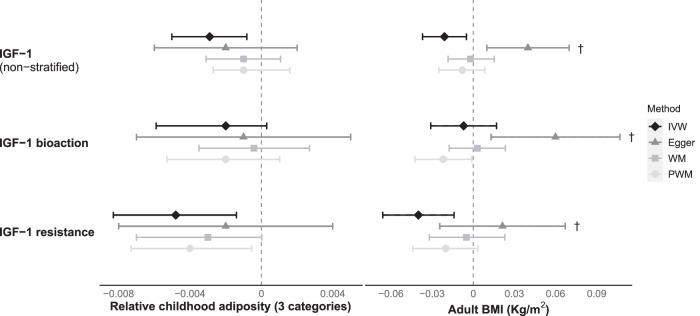
Estimated effects of IGF-1 (nmol/L) on relative childhood adiposity and adult BMI. IGF-1 bioaction was represented by IGF-1-increasing alleles also associated with taller childhood height. IGF-1 resistance was represented by IGF-1-increasing alleles also associated with shorter childhood height. Estimates are from random-effects inverse-variance weighted analyses with Steiger filtering. BMI body mass index, IGF-1 insulin-like growth factor-1, IVW inverse-variance weighted, WM Weighted Median, PWM Penalized Weighted Median. ^†^Indicates significant MR-Egger intercept (*P* < 0.05).

Overall, genetically-predicted higher IGF-1 concentrations were associated with lower relative childhood adiposity (β = −0.003 categories per nmol/L; *P* = 6.6 × 10^−3^) (Fig. 3 and Supplementary Table 3). Additional MR models using measured childhood BMI showed a directionally-consistent but non-significant association between higher IGF-1 and childhood BMI (*P* = 0.334) (Supplementary Fig. 2 and Supplementary Table 3). For adult BMI, IGF-1 showed directionally-inconsistent associations. The MR-Egger intercept indicated evidence of horizontal pleiotropy, and the MR-Egger estimate was in the opposite direction to estimates from the IVW, WM and PWM models (Fig. 3 and Supplementary Table 3).

#### IGF-1 bioaction

IGF-1-increasing alleles that were also associated with taller childhood height (indicative of greater IGF-1 secretion and bioaction) showed no association with relative childhood adiposity (β = −0.003 categories per nmol/L, *P* = 0.077) and adult BMI (β = −0.007 Kg/m^2^ per nmol/L, *P* = 0.562) (Fig. 3 and Supplementary Table 5). Additional MR models using measured childhood BMI showed no association with IGF-1 bioaction (*P* = 0.258) (Supplementary Fig. 2 and Supplementary Table 5).

#### IGF-1 resistance

Conversely, IGF-1-increasing alleles that were also associated with shorter childhood height (indicative of IGF-1 resistance) were associated with lower relative childhood adiposity (β = −0.005 categories per nmol/L; *P* = 6.7 × 10^−3^). Additional MR models using measured childhood BMI showed a similar negative association between IGF-1 resistance and childhood BMI (β = −0.016 SD per nmol/L; *P* = 9.5 × 10^−4^) (Supplementary Fig. 2 and Supplementary Table 5). For adult BMI, IGF-1 resistance showed directionally-inconsistent associations. The MR-Egger intercept indicated evidence of horizontal pleiotropy, and the MR-Egger estimate was in the opposite direction to estimates from the IVW, WM and PWM models (Fig. 3 and Supplementary Table 5).

## Discussion

This study examined how genetically predicted fasting insulin and IGF-1 affect childhood and adult adiposity to infer the causal effects of these biomarkers. Using a biologically informed approach, our findings indicate negative effects of insulin resistance on childhood and adult adiposity, as well as negative effects of IGF-1 resistance on childhood adiposity. We show that using a naive MR approach, which ignores the biological effects of biomarker-associated variants, we might wrongly conclude that fasting insulin and IGF-1 secretion and bioaction have negative effects on childhood and adult adiposity. Instead, our results highlight the importance of distinguishing between bioaction and resistance when analysing variants associated with biomarker levels.

For fasting insulin, recent MR analyses have suggested the importance of biologically-informed MR approaches to select appropriate instruments. Gagnon et al. filtered fasting insulin SNPs using data on pancreatic islet expression to identify 3 SNPs that they considered to represent increased insulin secretion and found a positive effect of these on BMI, even though there was no overall effect seen with all fasting insulin SNPs [22]. However, they did not model an effect of insulin resistance. In our study, we stratified fasting insulin SNPs according to their association with blood glucose levels, and we found a negative effect of insulin resistance on BMI, whereas there was no effect of insulin secretion on BMI. Similarly, the lack of a causal effect of insulin secretion on BMI aligns with an earlier analysis that filtered T2D-associated SNPs by their reported biological effects [23]. Our findings support the importance of biological filtering of SNPs in MR studies of biomarkers.

We observed a negative effect of IGF-1 resistance on childhood adiposity, but no consistent effect on BMI among adults aged 40–69 years (where the significant MR-Egger intercept indicates potential bias). These results may be consistent with cross-sectional findings showing a negative IGF-1-BMI relationship in the youngest quartile of that study (age 24–58 years) and a positive relationship in the oldest quartile (87–110 years), while no significant effect was observed among adults aged 58–86 years [24]. While the term ‘IGF-1 resistance’ is used here in a broad sense and does not specify any particular condition or biological pathway, IGF-1 resistance has been associated with a potential compensatory mechanism involving increased secretion of growth hormone (GH). Elevated GH levels have been found to have lipolytic effects [11]. Thus, IGF-1 resistance may indirectly contribute to the regulation of adiposity through the upregulation of GH secretion.

There are several limitations in our study. Firstly, the studied phenotypic traits as measures of adiposity may have limitations in fully capturing the complexity of body composition. We relied on self-reported, ordered, measures of childhood adiposity. However, the recalled childhood adiposity trait in UK Biobank was recently reported to show very high genetic correlation (r_g_ = 0.94) with objectively measured childhood BMI [13]. We also included as a sensitivity analysis data from a GWAS meta-analysis of childhood BMI, which supported most of our observed associations. Nevertheless, the use of ordered childhood obesity limits the interpretation of the effect estimates. Therefore, the effect sizes on childhood and adult adiposity could not be compared. Additionally, the biomarker measurements used in our study were from adults rather than children, and this MR is dependent on the assumption that the genetic determinants have consistent effects across the life-course. The reported genetic instruments for fasting insulin were from models adjusted for BMI, which could introduce collider bias [25]. However, this bias would affect all fasting insulin SNPs equally and does not explain the results of our biologically informed stratification. Our study was limited to European participants and the findings may not be applicable to other ethnicities. We also did not explore potential non-linear associations between IGF-1 and adiposity, as suggested by some phenotypic studies [6–10]. Finally, while we filtered biomarker-associated into two groups (representing bioaction or resistance), there is likely more complexity. Wang et al. used a statistical approach to separate IGF-1-associated SNPs into six clusters, although they were able to biologically annotate only two of these [26]. For example, it would be valuable in future to distinguish IGF-1 bioaction SNPs that act via increased GH secretion and signalling from those that act more directly.

In conclusion, this study provides new insights into the regulation of adiposity. Our findings indicate that insulin resistance has negative effects on childhood and adult adiposity, while IGF-1 resistance has negative effects on childhood adiposity. Furthermore, our study highlights the importance of employing “biological effect filtering” to differentiate between resistance and bioaction in MR studies, enhancing their interpretability and accuracy.

## Supplementary information

Supplementary figures

Supplementary tables

## Data Availability

Data from the Norwegian Mother, Father and Child Cohort Study and the Medical Birth Registry of Norway used in this study are managed by the national health register holders in Norway (Norwegian Institute of public health) and can be made available to researchers, provided approval from the Regional Committees for Medical and Health Research Ethics (REC), compliance with the EU General Data Protection Regulation (GDPR) and approval from the data owners. The consent given by the participants does not open for storage of data on an individual level in repositories or journals. Researchers who want access to data sets for replication should apply through helsedata.no. Access to data sets requires approval from The Regional Committee for Medical and Health Research Ethics in Norway and an agreement with MoBa.
