# Supplementary material for: Likely causal effects of insulin resistance and IGF-1 bioaction on childhood and adult adiposity: a Mendelian randomization study
Source: Int J Obes (Lond). 2024 Aug 22;48(11):1650–5. doi: 10.1038/s41366-024-01605-4 (PMC11502485; doi:10.1038/s41366-024-01605-4)
Supplement: Supplementary file 1 — Supplementary figures [file 41366_2024_1605_MOESM1_ESM.docx]

**Supplementary Figure 1.** **Estimated effects of fasting insulin (log_n_ pmol/L) on childhood BMI.** Insulin resistance was represented by fasting insulin-increasing alleles also associated with higher fasting glucose. Insulin bioaction was represented by fasting insulin-increasing alleles also associated with lower fasting glucose. Childhood BMI (SD) is based on 39,620 children aged between 2 and 18 years from the EGG consortium. Estimates are from random-effects inverse-variance weighted analyses with Steiger filtering.

BMI, body mass index; IVW, inverse-variance weighted; WM, Weighted Median; PWM, Penalized Weighted Median.

†Indicates significant MR-Egger intercept (*P*<0.05).

**Supplementary Figure 2.** **Estimated effects of IGF-1 (nmol/L) on childhood BMI.** IGF-1 bioaction was represented by IGF-1-increasing alleles also associated with taller childhood height. IGF-1 resistance was represented by IGF-1-increasing alleles also associated with shorter childhood height. Childhood BMI (SD) is based on 39,620 children aged between 2 and 18 years from the EGG consortium. Estimates are from random-effects inverse-variance weighted analyses with Steiger filtering.

BMI, body mass index; IGF-1, insulin-like growth factor-1; IVW, inverse-variance weighted; WM, Weighted Median; PWM, Penalized Weighted Median.

†Indicates significant MR-Egger intercept (*P*<0.05).
